# 1-methylhistamine as a potential biomarker of food histamine intolerance. A pilot study

**DOI:** 10.3389/fnut.2022.973682

**Published:** 2022-10-12

**Authors:** Sònia Sánchez-Pérez, Ricard Celorio-Sardà, M. Teresa Veciana-Nogués, M. Luz Latorre-Moratalla, Oriol Comas-Basté, M. Carmen Vidal-Carou

**Affiliations:** ^1^Departament de Nutrició, Ciències de l’Alimentació i Gastronomia, Facultat de Farmàcia i Ciències de l’Alimentació, Campus de l’Alimentació de Torribera, Universitat de Barcelona, Santa Coloma de Gramenet, Spain; ^2^Institut de Recerca en Nutrició i Seguretat Alimentària (INSA-UB), Universitat de Barcelona, Santa Coloma de Gramenet, Spain; ^3^Xarxa d’Innovació Alimentària, Barcelona, Spain

**Keywords:** histamine, 1-methylhistamine, histamine intolerance, diamine oxidase (DAO), DAO deficiency, food intolerance

## Abstract

Efforts are currently being directed to identify a non-invasive marker that can serve as a solid and clinically irrefutable diagnostic criterion for histamine intolerance associated with diamine oxidase (DAO) deficiency. Accordingly, the identification of biomarkers of histamine (HA) metabolism in urine is proposed as a possible new diagnostic strategy. It is hypothesized that individuals with histamine intolerance could have a different urinary profile of HA and its metabolites in comparison with the healthy population. Thus, the aim of this study was to assess the urinary excretion of HA and 1-methylhistamine (MHA) in individuals diagnosed with histamine intolerance and in a control group. Levels of HA and MHA were compared between 24 h and first morning spot urine in a subgroup of 14 control individuals. Then, HA and MHA concentrations in spot urine of 32 histamine intolerant and 55 control individuals were determined by ultra-high performance liquid chromatography and fluorometric detection (UHPLC-FL) and normalized by creatinine. No differences were found between HA and MHA levels in 24 h and first morning samples. Overall, histamine intolerant patients presented a distinct urinary excretion profile compared to the control group due to lower levels of MHA. No differences in urinary MHA were observed related to serum DAO activity. Spot urine samples were thus validated as a reliable tool to determine the urinary excretion of HA and MHA. These results constitute a starting point for the study of HA metabolomics as a suitable and non-invasive approach to histamine intolerance diagnosis.

## Introduction

Histamine (2-[4-imidazolyl] ethylamine), a bioactive amine with important physiological activities in the organism, can be found in many frequently consumed foods (1, 2). Dietary histamine (HA) is mainly formed by bacterial decarboxylation of the amino acid histidine. It is found especially in microbiologically spoiled or fermented products, generated by the enzymatic activity of the contaminating or technological bacteria (1, 3).

In healthy people, and under normal conditions, HA is metabolized by two main pathways. One involves oxidative deamination by the enzyme diamine oxidase (DAO) to form imidazole acetaldehyde, which is subsequently transformed into imidazoleacetic acid (IAA) by the action of aldehyde dehydrogenase (ALDH) and combined with a ribose for its excretion. Alternatively, the action of histamine-N-methyltransferase (HNMT) produces 1-methylhistamine (MHA), which is converted by ALDH to N-methylimidazoleacetic acid (MIAA) (2, 4). HNMT is expressed in almost all tissues, while DAO is active mainly in the intestines, kidneys, and placenta. At the intestinal level, DAO plays a key role in the degradation of histamine from food and regulates its passage into the systemic circulation (2, 4).

Histamine intolerance is a disorder arising from reduced histamine degradation capacity in the intestine due to impaired DAO activity, leading to its accumulation in plasma and the appearance of adverse effects. DAO deficiency may be inherited or due to inflammatory and degenerative intestinal disorders, the intake of DAO-blocking drugs or intestinal dysbiosis (5, 6). Histamine intolerant individuals usually show symptoms in two or more non-specific organs or systems, such as gastrointestinal tract (e.g., bloating, diarrhea, abdominal pain, postpandrial fullness, constipation, flatulences, and nausea) nervous system (e.g., headache and dizziness), cardiovascular system (e.g., tachycardia, hypotonia, and collapse), skin (e.g., pruritus, eczema, urticaria, edemas, and flushing) and respiratory system (e.g., rhinorrhea, rhinitis, nasal congestion, and dyspnea) (7). Preventing the onset of symptoms by dietary management involves following a low-histamine diet and supplementation with exogenous DAO (8, 9).

Histamine intolerance is currently diagnosed using a combination of criteria, including the absence of other potential causes of higher HA levels in the organism (i.e., food allergy and mastocytosis) and the presence of two or more typical symptoms, which improve or remit after following a low-histamine diet (5, 10–12). However, there is not a clear consensus in the diagnostic criteria for histamine intolerance. Frequently the diagnosis is complemented by the determination of serum DAO activity, although the evidence for the validity of this measurement is neither abundant nor conclusive (12–17). In recent years, efforts have been made to identify a non-invasive marker that could serve as a solid and clinically irrefutable diagnostic criterion for DAO-related histamine intolerance. In this context some studies suggest the used of the determination of single-nucleotide polymorphisms related to a lower DAO activity, as well as the identification of biomarkers of histamine metabolism in urine as possible new diagnostic strategies (18). The hypothesis is that individuals with histamine intolerance may have a different excretion profile of HA and its metabolites in urine in comparison with healthy individuals. In a previous study, Comas-Basté et al. developed a Ultra-High Performance Liquid Chromatography (UHPLC) method that allows rapid and accurate analysis of HA and MHA in human urine (18). However, data for the potential diagnostic utility of the HA metabolomic profile in urine to identify histamine intolerant phenotypes are still lacking. Thus, the aim of this study was to analyze the urinary excretion of HA and MHA in individuals with symptoms compatible with histamine intolerance in comparison with a control group. Additionally, the behavior of HA and MHA urinary levels during 9 months of dietary treatment of histamine intolerance was studied in a smaller subgroup of patients.

## Materials and methods

### Study design and participants

A total of 87 adult volunteers participated in this study. Individuals with histamine intolerance (*n* = 32, mean age = 43.5 ± 8.8 years, 100% women) were recruited from a nutrition and dietetic center specialized in the dietary management of DAO deficiency (DAO Deficiency Clinical Institute, Barcelona, Spain). All the recruited patients keen to participate were women. In general, histamine intolerance is a clinical frame with a higher prevalence in women (19). The inclusion criteria for the histamine intolerant patients were as follows: aged between 18 and 65 years; diagnosis of histamine intolerance according to Mušič, et al. based on two or more symptoms; and negative results for food allergen-specific IgE (12). The exclusion criteria were pregnancy, lactation, having started a low-histamine diet and having taken antibiotics and/or probiotics the month before the study. Sixteen out of 32 patients with symptoms compatible with histamine intolerance also had plasma DAO deficiency, measured using a radio extraction assay according to the manufacturer’s instructions (Sciotec Diagnostic Technologies, Tulln, Austria).

The individuals in the control group (*n* = 55, mean age = 32.3 ± 5.7 years, 64% women) were volunteers recruited at the Food and Nutrition Campus of the University of Barcelona (Santa Coloma de Gramenet, Barcelona) with no symptoms associated with histamine intolerance (total absence or the presence of one symptom at most) and without other food intolerances or allergies.

As a preliminary phase, the excretion of HA and MHA was analyzed in urine samples collected over 24 h and in spot samples (first morning urine) in a subgroup of 14 healthy volunteers. For the analysis of urinary HA and MHA, spot samples of every participant were collected without the addition of preservatives and kept under refrigeration until handled in the laboratory. Samples were stored at −80^°^C until their analysis. Additionally, the urinary excretion of HA and MHA in a subgroup of five histamine intolerance patients with DAO deficiency (< 10 U/ml) was monitored during 9 months of dietary treatment (sampling points: 2, 6, and 9 months). The first 2 months of the dietary management of histamine intolerance were the most restrictive, with the exclusion of all histamine-rich foods and oral supplementation with exogenous DAO. Thereafter, excluded foods were gradually introduced according to the tolerance of the patient, and DAO supplementation was still administered. Recommendations given to the patients on how to follow a low-histamine diet are shown in Supplementary Table 1. These recommendations include foods that are likely to contain histamine and those that, although they do not contain histamine, may contain other biogenic amines (8). The presence of these other amines can exert an inhibitory effect on the histamine degradation by DAO enzyme due to the competition for this degradation system (20).

All volunteers received a kit consisting of an information sheet detailing the purpose and conditions of the study, an informed consent document, a questionnaire for data collection and a urine sample collection protocol. The study was approved by the Ethics Committee of the University of Barcelona (Institutional Review Board IRB00003099).

### Determination of urinary histamine and 1-methylhistamine by ultra-high performance liquid chromatography and fluorometric detection

Histamine and MHA levels in urine were analyzed as described by Comas-Basté et al. (18). Briefly, the analytical method consists of sample preparation, purification, and concentration by the acidic hydrolysis of urine samples followed by solid phase extraction using mixed cation exchange cartridges. Overall, the analytical procedure allowed for a 50-fold concentration of the analytes in relation to the initial urine content. Chromatographic separation of HA and MHA was performed using UHPLC and a BEH C18 chromatographic column (1.7 μm, 2.1 mm × 50 mm) (Acquity™, Waters Corp., Milford, MA, USA). Fluorometric detection of both analytes was achieved through an online post-column derivatization system using o-Phthalaldehyde (OPA) as the fluorescence reagent. Quantification of HA and MHA levels in urine was accomplished by the external standard procedure, using histamine dihydrochloride and 1-methylhistamine dihydrochloride as the analytical standards (Sigma, St. Louis, MO, USA).

### Determination of urinary creatinine

Excreted HA and MHA levels were normalized based on urinary creatinine concentration. This is the recommended approach when estimating the levels of biological substances eliminated in specific urine samples due to the relative stability of creatinine in the absence of renal pathologies (21). Thus, to normalize urinary excretion values, creatinine in urine was determined using the colorimetric alkaline picrate method (Jaffé reaction) adapted to microplates (Thermo Scientific™ 96-Well Microtiter Microplates), as described in Medina-Remón et al. (22). Briefly, 3 μL of urine was mixed with 60 μL of aqueous picric acid solution (1%) and 5 μL of sodium hydroxide (10%). The mixture was stirred and allowed to stand for 15 min in darkness at room temperature. Subsequently, 232 μL of Milli-Q water was added and the absorbance was read at 500 nm on a UV/VIS spectrophotometer. The concentration of HA and MHA in urine was expressed in μg of analyte per g of creatinine.

The excretion of creatinine in urine was within the normal values for all study participants and no significant differences were found between the mean excretion values of the two study groups (*p* > 0.05) (23).

### Statistical analysis

The statistical analysis of data was performed with Statistical Software Package for Windows SPSS, version 25.0 (SPSS, Chicago, IL, USA). Student’s *t*-test for paired and unpaired samples and the Mann–Whitney *U* Test for non-parametric data were used to assess the significance of differences between urine samples. Pearson’s Correlation Coefficient was also used to estimate the goodness of the correlation between 24 h urine samples and specific urine samples.

## Results

### Reliability of spot urine sample analysis

Spot urine (first morning urine) is the most commonly used type of urine sample in clinical studies, as collecting 24 h urine samples is not patient-friendly. For this reason, in the preliminary phase of the current study, the reliability of spot urine samples was evaluated by comparing the urinary excretion of HA and MHA in spot and 24 h urine samples of 14 subjects.

**FIGURE 1:**
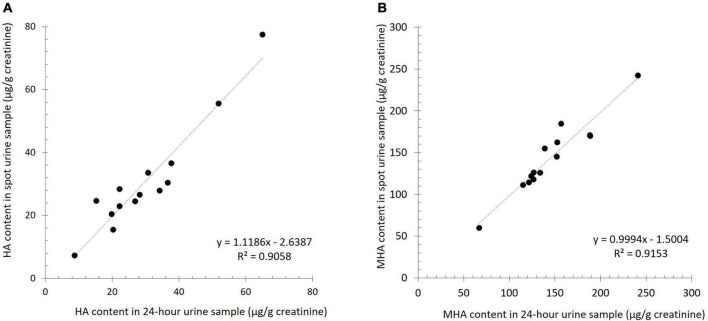
Linear correlation for the urinary excretion of histamine (HA, **A**) and 1-methylhistamine (MHA, **B**) between spot urine samples and 24 h urine samples.

The mean HA concentration in spot and 24 h samples was 30.9 ± 17.3 μg/g creatinine and 30.0 ± 14.7 μg/g creatinine, respectively and the mean MHA excretion was 143.5 ± 43.0 μg/g creatinine, and 145.1 ± 41.1 μg/g creatinine, respectively. No significant differences were detected in the urinary excretion of HA (*p* > 0.05) and MHA (*p* > 0.05) between the two sample types of each individual. In fact, a high correlation was found between the urinary content of HA and MHA between the spot and 24 h urine samples (*r* = 0.952, *r* = 0.957, respectively) (Figure 1).

### Urinary histamine and 1-methylhistamine profile in control and histamine intolerant individuals

Patients with histamine intolerance showed an average of 4.5 ± 1.1 symptoms and 78% of them reported combinations of more than three symptoms. The most frequent complaints were gastrointestinal (94% of the patients), mainly bloating, flatulence and abdominal pain, followed by some dermatological manifestations (74%), such as urticaria and atopic skin. More than 80% of patients reported suffering from a simultaneous combination of gastrointestinal and dermatological symptoms. Neurological and respiratory system affections were also reported, although to a lesser extent. Lower serum DAO activity was not correlated with a higher number of symptoms.

**FIGURE 2:**
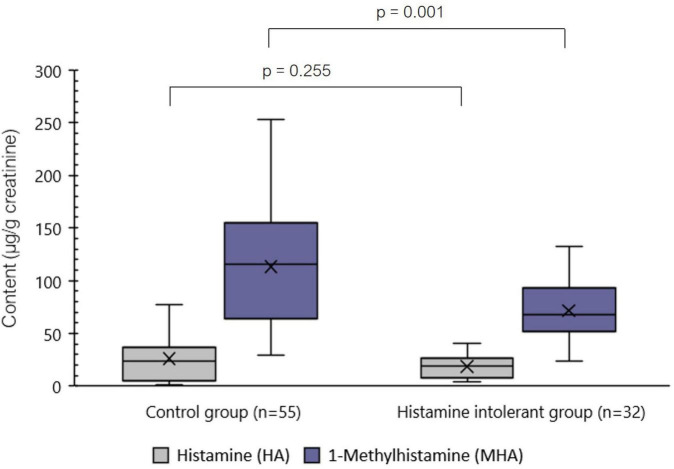
Distribution of the levels (μg/g creatinine) of histamine (HA) and 1-methylhistamine (MHA) in spot urine samples of control and histamine intolerant individuals. *p* < 0.05 indicates statically significant differences in the urinary content of the analytes between study groups.

Figure 2 shows the distribution of urinary HA and MHA levels in the control and histamine intolerant groups. The values of the two analytes varied widely in both study groups, especially in the case of MHA, which had an interquartile range (IQR = P75–P25) of 85.15 μg/g creatinine and 35.83 μg/g creatinine for the control and histamine intolerant groups, respectively. Histamine levels were not significantly different between the two groups, with mean values of 18.65 ± 11.08 μg/g creatinine for the histamine intolerant group and 26.25 ± 22.17 μg/g creatinine for the control (*p* > 0.05). In contrast, MHA values were significantly lower in patients with symptoms of histamine intolerance (70.29 ± 29.43 μg/g creatinine) compared with the control (112.99 ± 55.48 μg/g creatinine) (*p* < 0.01).

Given that the composition of the two study groups was not homogeneous in terms of sex (the histamine intolerant group consisted entirely of women), potential differences in analyte excretion levels between the sexes were analyzed within the control group. Accordingly, no significant differences in HA and MHA concentration were observed between healthy men and women (*p* > 0.05).

**FIGURE 3:**
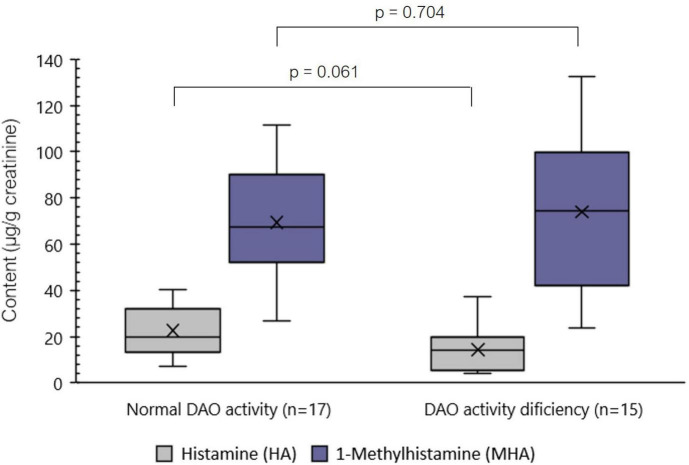
Distribution of the levels (μg/g creatinine) of histamine (HA) and 1-methylhistamine (MHA) in spot urine samples of individuals with symptoms of histamine intolerance with or without serum DAO enzyme deficiency. *p* < 0.05 indicates statically significant differences in the urinary content of the analytes between study groups.

When stratifying individuals with symptoms of histamine intolerance based on the presence or absence of deficient serum DAO activity, no statistically significant differences were found for the levels of urinary HA (*p* > 0.05) or MHA (*p* > 0.05) (Figure 3). Moreover, compared with the control group, individuals with deficient serum DAO activity showed similar levels of urinary HA (*p* > 0.05) but significantly lower MHA levels (*p* < 0.05). The same trend was observed when considering the whole histamine intolerant group.

### Influence of the dietary treatment of histamine intolerance on the urinary excretion of histamine and 1-methylhistamine

**FIGURE 4:**
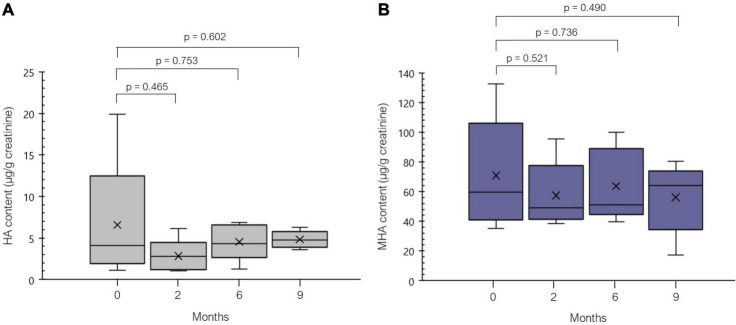
Distribution of the levels of (μg/g creatinine) of histamine (HA, **A**) and 1-methylhistamine (MHA, **B**) during 9 months of dietary treatment (low-histamine diet and DAO enzyme supplementation) in five patients.

A small-sized study on a subgroup of five patients with symptoms of histamine intolerance was performed to assess the influence of the dietary treatment of histamine intolerance on the urinary excretion of HA and MHA (Figure 4). At the initial sampling point (time 0), before the start of the dietary treatment, considerable variability was observed in HA urinary levels (IQR = 10.54 μg/g creatinine).

After 2 months of dietary treatment (i.e., a highly restrictive exclusion of HA-containing foods together with oral supplementation with DAO), a reduction of the mean HA and MHA excretion values was observed, although without statistical significance (*p* > 0.05). At this point, two patients showed marked reductions in the urinary excretion of HA and MHA (up to 86 and 56%, respectively). Thereafter, the urinary excretion of both analytes remained quite constant for all five patients (*p* > 0.05).

## Discussion

Early-morning spot urine was found to be suitable for the analysis of urinary HA and MHA, as the content of both analytes was highly correlated with the levels in 24 h samples. This supports the findings of other authors (24, 25), who report that the analyte levels in a spot urine sample correlate with a 24 h collection as long as creatinine values are used to normalize the excretion values. Moreover, Saito et al. found a wide variability in urinary HA values throughout the day and indicated the usefulness of normalizing them as a ratio to creatinine to compensate for these fluctuations (25). As well as their convenience for patients, early-morning urine samples may provide more reliable results due to a higher analyte concentration (including creatinine) (26). Nevertheless, infectious bacterial strains in the urinary tract, especially in women, may potentially influence HA levels in early-morning samples.

In this study, urinary HA excretion values in histamine intolerant and control individuals were very similar (mean values of 18.65 ± 11.08 μg/g creatinine and 26.25 ± 22.17 μg/g creatinine, respectively, *p* > 0.05). Additionally, within the histamine intolerant group, no significant differences were found in excreted HA between individuals with and without deficient serum DAO activity (*p* > 0.05). In all cases, the HA values observed in the different individuals of both study groups fell within the ranges previously described by Keyzer et al. (9.8–73.6 μg/g creatinine) and Oosting et al. (8.8–55.9 μg/g creatinine) in healthy individuals (26, 27). Likewise, Lamale et al. reported normal HA excretion levels in healthy adult women, ranging between 22.4 and 58.2 μg/g creatinine, but found a significant increase in women with interstitial cystitis (28).

In contrast, we observed significant differences in the urinary concentration of MHA, the levels being markedly lower in histamine intolerant individuals (71.58 ± 29.43 μg/g creatinine) compared to the control population (112.99 ± 55.48 μg/g creatinine) (*p* < 0.05). Within the histamine intolerant group, urinary MHA values were very similar between those with or without deficient serum DAO activity (*p* > 0.05); moreover, there was no apparent relationship between levels of urinary MHA and the frequency or type of symptomatology. Regardless of the differences observed between groups, the urinary MHA excretion values of most individuals in both groups match those reported by several authors in previous studies (55–230 μg/g of creatinine) (24, 27, 29). This great variability in MHA excretion could be partially explained by interindividual differences in the functionality of HA degradation systems in the organism (29).

The observed reduction in MHA levels in the histamine intolerant group indicates that the urinary HA and MHA profile could be a more reliable biomarker of histamine intolerance than serum DAO activity. In this study, only 50% of patients diagnosed with histamine intolerance based on the presence of symptoms were identified by DAO activity measurement.

As MHA is an HA-derived metabolite arising from the action of the enzyme HNMT, lower MHA levels in histamine intolerant individuals with DAO deficiency were not expected. A possible explanation is that impaired DAO activity could lead to a compensatory activation of HA metabolism by HNMT (30, 31), and ultimately an increase in the pathway end-product (i.e., MIAA). Thus, as well as potentially confirming the current results, the measurement of IAA and MIAA levels in urine would provide a more complete profile of HA excretion and its possible alteration in histamine intolerant individuals. New insight into the HA metabolome in this population would also be obtained.

Besides the use of the HA and MHA as a potential biomarker for histamine intolerance, a smaller preliminary study was performed with a subgroup of five histamine intolerance patients, in order to assess the behavior of HA and MHA urinary levels during 9 months follow-up of a low-histamine diet and oral DAO supplementation. To date, only a few studies have assessed the effect of dietary HA on the urinary levels of HA and its metabolites and they have been focused on healthy individuals (32–35). For example, in the study performed by Granerus, histamine was given orally to three healthy subjects, resulting in higher levels of urinary HA and the metabolites MHA and especially MIAA. Moreover, the urinary excretion of MIAA was found to be a dose-dependent on the administration of exogenous HA (32). Similarly, Keyzer et al. reported an increase in the excretion of MIAA after the oral administration of 200 μM of HA in three healthy individuals, with no significant changes in MHA. A high correlation was also found between protein intake and the urinary excretion of HA metabolites (33). According to our knowledge the current work is the first study with histamine intolerant patients. The results of the current study revealed a decrease in the mean urinary levels of HA and MHA after the first 2 months, although without statistical significance. The reduction was especially marked in two patients. These results can be explained by the highly restrictive nature of the diet at the beginning of the treatment, when a wide range of foods with typically high HA contents were excluded (e.g., fermented foods and beverages, preserved and semi-preserved fish products, spinach, eggplant, tomato, citrus, and nuts) (8). No clear trend was observed for HA and MHA levels during the reintroduction phase of the dietary treatment. The determination of a wider range of metabolites, including MIAA, could allow a more accurate assessment of the influence of the dietary treatment of histamine intolerance on the urinary profile of HA in patients with histamine intolerance.

## Limitations

The main limitations of the present study were the rather small size of each sample group and the lack of male representation in the histamine intolerance group (although no differences were found between men and women in the control group). Moreover, it would have been of interest to measure the intensity of the clinical symptoms of the patients to elucidate any correlation with the urinary HA profile. Lastly, it is important to highlight that the preliminary study on the influence of a dietary treatment of histamine intolerance on the urinary excretion of HA and MHA was only assessed in five individuals, thus limiting the extrapolation of the results.

## Conclusion

To the best of our knowledge, this is the first time that the determination of urinary HA and its metabolites, particularly MHA, is proposed as a diagnostic tool for histamine intolerance to complement the current criteria based on the assessment of symptoms. The validity of using spot urine samples to measure the urinary excretion of HA and MHA has been confirmed. According to the obtained results, the hypothesis that the excretion profile in individuals with symptoms of histamine intolerance differed from that of the healthy individuals was demonstrated due to lower urinary levels of MHA in histamine intolerant patients. However, no differences were observed in urinary MHA related to the level of serum DAO activity. These results constitute a starting point for the study of HA metabolomics as a suitable and non-invasive approach to histamine intolerance diagnosis. An area for further research is the influence of a low-histamine diet and DAO supplementation on the urinary excretion of HA in histamine intolerant patients.

## Data availability statement

The raw data supporting the conclusions of this article will be made available by the authors, without undue reservation.

## Ethics statement

The studies involving human participants were reviewed and approved by the Ethics Committee of the University of Barcelona (Institutional Review Board IRB00003099). The patients/participants provided their written informed consent to participate in this study.

## Author contributions

MV-N, ML-M, and MV-C: conceptualization. SS-P, RC-S, and OC-B: investigation and writing – original draft preparation. SS-P, RC-S, MV-N, ML-M, OC-B, and MV-C: writing – review and editing. OC-B and MV-C: supervision. All authors have read and agreed to the published version of the manuscript.
