# Supplementary material for: 1-methylhistamine as a potential biomarker of food histamine intolerance. A pilot study
Source: Front Nutr. 2022 Oct 12;9:973682. doi: 10.3389/fnut.2022.973682 (PMC9597364; doi:10.3389/fnut.2022.973682)
Supplement: Supplementary file 1 [file Table_1.docx]

| **Foods** | **Excluded Foods** | **Allowed Foods** |
| --- | --- | --- |
| *Cereals and tubers* | - | Rice, oats, spelt, corn, millet, quinoa, wheat, buckwheat, sweet potato, potato, cassava, etc. |
| *Fruits and nuts* | Strawberries and red fruits, papaya, avocado, citrus fruits (orange, grapefruit, tangerine, lemon, kiwi, pineapple), banana, plums, nuts (walnuts, peanuts, almonds, cashews, hazelnuts) | Apricot, cherries, pomegranate, fig, mango, apple, peach, melon, pear, grape, watermelon, pine nuts, etc. |
| *Vegetables* | Vegetable pickles (p.ej. sauerkraut, olives), zucchini, pumpkin, spinach, eggplant, tomato) | Chard, artichoke, broccoli, onion, fennel, green beans, asparagus, lettuce, red and green pepper, beet, carrot, etc. |
| *Dairy products and substitutes* | Cured, semi-cured and grated cheeses,  milk and yogurts | Fresh cheeses, vegetable drinks, fermented products based on soy or other vegetables (“vegetable yogurt”) |
| *Legumes and derivative*s | Sufu, Tempeh | Beans, kidney beans, chickpeas, lentils, soybeans, etc. |
| *Fish and derivatives* | Seafood, canned or semi-canned fish (canned tuna, anchovies, canned sardines or mackerel, anchovies in vinegar, smoked salmon, etc.) | White or blue fish, cuttlefish, squid, octopus, etc., fresh or frozen* |
| *Egg* | Egg white | Yolk |
| *Meat and poultry* | Dry-fermented sausages (fuet, spicy pork sausage, Iberian spicy sausage...), viscera (liver, foie-gras, kidney, etc.) | Fresh or frozen meat*, cured ham or loin, cooked ham or turkey, mortadella, Frankfurt, etc. |
| *Drinks* | Orange juice, tomato juice, tea, alcoholic beverages (cava, wine, beer, cider, spirits), | Water, infusions, coffee, etc. |
| *Fats* | - | Olive oil and seeds, toasted sesame cream (tahin), butter and margarine |
| *Condiments* | Vinegar, soy sauce, tomato sauce | Oregano, basil, turmeric, ginger, mint, salt, sugar, honey and sweeteners |
| *Others* | Chocolate, citrus jam,  bakery and pastry products containing excluded foods | Carob, compotes and suitable fruit sorbets, chia and flax seeds, sunflower and pumpkin |

*It will be important to ensure that both fish and meat are very fresh at the time of purchase. Otherwise it will be preferable to buy them directly frozen. Data from Sanchez-Perez et al., 2021 (8)
